# Both extracellular vesicles from helicobacter pylori-infected cells and helicobacter pylori outer membrane vesicles are involved in gastric/extragastric diseases

**DOI:** 10.1186/s40001-023-01458-z

**Published:** 2023-11-06

**Authors:** Chengyao Wang, Wenkun Li, Linlin Shao, Anni Zhou, Mengran Zhao, Peng Li, Zheng Zhang, Jing Wu

**Affiliations:** Department of Gastroenterology National Clinical Research Center for Digestive Disease, Beijing Digestive Disease Center, BeijingKey Laboratory for Precancerous Lesion of Digestive Disease, Beijing Friendship Hospital, Capital Medical University, Beijing, 100050 People’s Republic of China

**Keywords:** Extracellular vesicles, Outer membrane vesicles, Helicobacter pylori, Gastric cancer, Extra-gastrointestinal disease

## Abstract

Bacterial-derived extracellular vesicles (EVs) have emerged as crucial mediators in the cross-talk between hosts and pathogens, playing a significant role in infectious diseases and cancers. Among these pathogens, Helicobacter pylori (*H. pylori*) is a particularly important bacterium implicated in various gastrointestinal disorders, gastric cancers, and systemic illnesses. *H. pylori* achieves these effects by stimulating host cells to secrete EVs and generating internal outer membrane vesicles (OMVs). The EVs derived from *H. pylori*-infected host cells modulate inflammatory signaling pathways, thereby affecting cell proliferation, apoptosis, cytokine release, immune cell modification, and endothelial dysfunction, as well as disrupting cellular junctional structures and inducing cytoskeletal reorganization. In addition, OMVs isolated from *H. pylori* play a pivotal role in shaping subsequent immunopathological responses. These vesicles incite both inflammatory and immunosuppressive reactions within the host environment, facilitating pathogen evasion of host defenses and invasion of host cells. Despite this growing understanding, research involving *H. pylori*-derived EVs remains in its early stages across different domains. In this comprehensive review, we present recent advancements elucidating the contributions of EV components, such as non-coding RNAs (ncRNAs) and proteins, to the pathogenesis of gastric and extragastric diseases. Furthermore, we highlight their potential utility as biomarkers, therapeutic targets, and vehicles for targeted delivery.

## Introduction

Helicobacter pylori (*H. pylori*) is a spiral-shaped, spiky, gram-negative bacterium found in the gastric tract of about half the world's people [1]. This bacterial infection has been linked to the pathogenesis of various gastrointestinal disorders, including gastritis, stomach ulcers, MALT (mucosa-associated lymphoid tissue) lymphomas, and stomach cancer [2]. *H. pylori* thrives in the stomach due to the unique environment provided by the gastric tissue barrier. It can directly injure superficial epithelial cells or stimulate the release of proinflammatory mediators from these cells. Additionally, *H. pylori*-derived products can penetrate the underlying mucosa, triggering both non-specific and specific immune responses in the host [3]. The literature also implicated this gastric pathogen in the development of extra-digestive diseases, such as atherosclerosis (AS) [4], chronic obstructive pulmonary disease (COPD) [5], nonalcoholic fatty liver disease (NAFLD) [6], blood diseases (idiopathic thrombocytopenic purpura, iron deficiency anemia) [7], inflammatory bowel disease (IBD) [8], skin diseases [9], and Alzheimer's disease (AD) [10]. Although infection with *H. pylori* has been shown to cause leaky bowel by impairing the tight-junctional proteins occludin, claudin-4, and claudin-5, there is little evidence of *H. pylori* in the blood [11]. It has been suggested that extra-gastric symptoms result from insulin resistance concerning proinflammatory cytokines produced by inflamed mucosa and acute phase reactants [12]. However, the fundamental mechanism by which *H. pylori* products cross the epithelial barriers to affect other systems via the bloodstream remains unclear.

Extracellular vesicles (EVs) are membrane particles with a lipid bilayer surrounding a cytosol compartment [13]. EVs are rich in bioactive molecules, including lipids, proteins, and nucleic acids (DNA, mRNAs, microRNAs, and other non-coding RNAs) [14]. Unlike direct cell-to-cell contact with signaling molecules secreted by cells, EVs are widely recognized as a novel intercellular messenger within the body. They can influence surrounding cells by either immediately releasing material from the vesicle or transporting contents from the donor to the recipient cell [15]. In infectious diseases, infected cells release EVs, and viruses, bacteria, parasites, and fungi also release EVs during infection. These EVs contain factors derived from both the pathogen and the host, and they play a pivotal role in pathogen uptake, replication, and regulation of the host immune response [16]. In recent years, accumulating evidence has shown that EVs are essential in regulating diverse cellular activities in *H. pylori*-related diseases. They can be released by host cells or bacteria and transport biological signaling molecules [17]. This review aims to present a comprehensive overview of the association between *H.pylori* infection and gastric/extra-gastric diseases. We will focus on investigating the involvement of EV-ncRNA/protein components in the development and progression of *H. pylori*-associated diseases. Additionally, we will explore the potential of these EV components as biomarkers, therapeutic targets, and delivery vehicles in diverse host systems.

### Classification and biology of extracellular vesicles

**Fig. 1:**
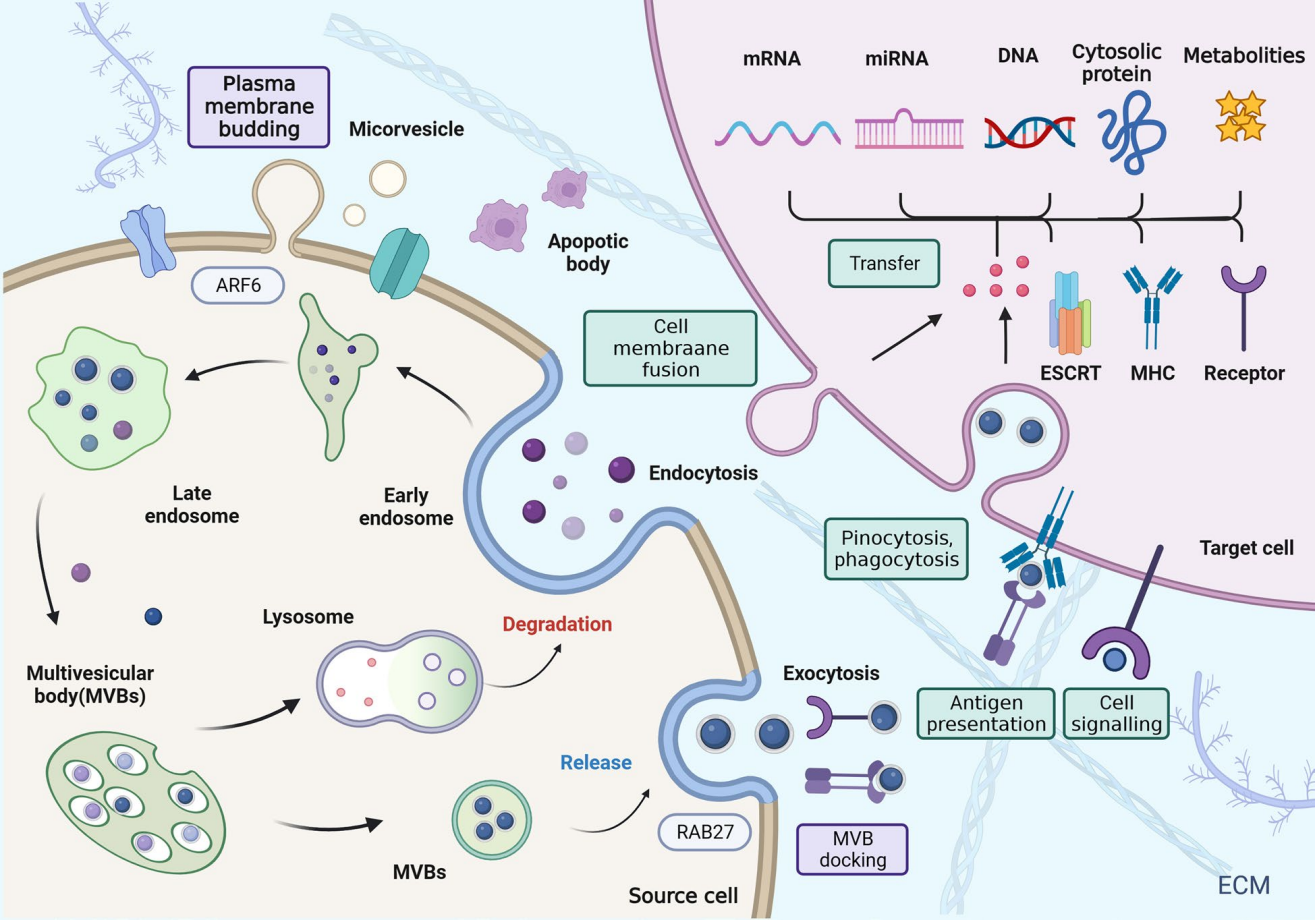
The biogenesis of EVs. The cytoplasmic membrane first invaginates exosomes to form early endo nucleosomes, which further develop into late endo nucleosomes, and then the endo nucleosome membrane invaginates to form multivesicular bodies (MVBs). MVB are produced in two ways. On one hand, they can fuse with lysosomes and be degraded. On the other hand, MVBs fuse with the cell membrane and release their encapsulated luminal vesicles into the extracellular space, thus forming exosomes. The exosomes are released and can interact with the recipient cell via cell signaling molecules on their surface. Exosomes can also act by different mechanisms such as endocytosis, phagocytosis or direct fusion with the cell membrane into the recipient cell. Microvesicles are formed through direct outward budding and shedding from the plasma membrane. Apoptotic bodies(ABs) are small membranous particles released during programmed cell death

Based on size and biology, the International Society for Extracellular Vesicles (ISEV) classifies EVs into three basic subgroups: exosomes, microvesicles (MVs), and apoptotic bodies (ABs) [18]. Microvesicles, with sizes ranging from 100 to 1000 nm, are generated through direct budding of the plasma membrane. ABs are bilayer lipid vesicles formed during programmed cell death by plasma membrane vesicles ranging from 1000 to 5000 nm [19, 20]. Exosomes, which have a size range of 30–150 nm, are produced and released by many different cells; they are endosomal in origin and released from multivesicular bodies (MVBs) into the extracellular space involving an endosomal sorting required for transport (ESCRT) machinery or the ESCRT independent pathway [21–23] (Fig. 1). The ESCRT machinery, consisting of four complexes (ESCRT-0, -I, -II, and -III) and associated proteins, is crucial for endosomal sorting and membrane remodeling. Its function involves identifying and binding to cargo that is ubiquitinated (ESCRT-0), concentrating cargo, and recruiting ESCRT-III, specifically ESCRT-I and -II. This process promotes membrane budding and fission, resulting in the formation of intraluminal vesicles (ILVs) within multivesicular bodies (MVBs). In addition, an ESCRT-independent pathway exists that employs distinct mechanisms for cargo sorting and trafficking. This pathway involves membrane bulging and fission mediated by lipid microdomains, which require the participation of accessory proteins and enzymes [24–27]. Various factors, such as cargo characteristics and cellular context, influence vesicle sorting and formation. Pathways and molecules involved in signaling activate and regulate specific sorting mechanisms. Pathological conditions can disrupt the sorting machinery and affect pathway preference, impacting vesicle trafficking. It’s important to note that the ESCRT machinery and ESCRT-independent pathways can interact and coordinate depending on cargo and cellular context, ensuring efficient sorting and cellular homeostasis [28–30].

In addition to inducing host cells to produce EVs, *H. pylori* can also secrete bacterial extracellular vesicles (bEVs), commonly known as outer membrane vesicles (OMVs), by shedding its outer membrane [31]. The formation of OMVs (released by *H. pylori*), which contain various constituents of the bacteria such as proteins, toxins, phospholipids, and nucleic acids, is now considered a means of transferring pathogenic factors from bacteria to host cells [32]. It is essential for the structure of the cell envelope, metabolism, communication between bacteria, and the formation of biofilms. Notably, Ricci et al. [33] demonstrated that vacuolating cytotoxin (VacA), when encapsulated in OMVs, can serve a distinct function from free lytic toxins. For example, OMVs can act as an alternative delivery system in environments other than the stomach lining (intestine), where OMVs interact with epithelial cells and potentially disrupt their integrity. The precise mechanism by which pathogenic factors enter host cells through OMVs remains incompletely understood. Chew et al. [34] utilized confocal microscopy to fluorescently label OMVs and observed that *H. pylori* OMVs predominantly enter the Human Gastric Adenocarcinoma (AGS) Cell through macrophage phagocytosis, ruling out direct fusion between OMVs and the AGS cell membrane. Moreover, there is a lack of consensus regarding the precise mechanisms through which OMVs from the same pathogen can invade non-phagocytic host cells, resulting in an unresolved understanding of the exact mode of OMV host cell entry [35].

### Nucleic acid components of extracellular vesicles

Among the different EV cargos, ncRNAs are one of the most abundant [36]. NcRNAs act as RNA molecules transcribed from the genome and do not encode proteins that play essential roles in activating and silencing genes, regulating transcription, and splicing and modifying RNA [37]. NcRNAs in EVs employ various mechanisms to evade degradation both in the extracellular environment and within recipient cells. The lipid bilayer membrane enclosing EVs serves as a physical barrier, shielding the ncRNAs against RNA-degrading enzymes present in the extracellular milieu. Moreover, ncRNAs interact with RNA-binding proteins, forming ribonucleoprotein complexes (RNPs) that confer stability and protection. Some ncRNAs also adopt specific secondary structures that make them less susceptible to degradation enzymes. Upon uptake by recipient cells, ncRNAs may associate with intracellular RNA-binding proteins or reside in specialized compartments, such as endosomes or P-bodies, that modulate RNA stability [38–40].

Regulatory ncRNAs can be classified by length (< 200 or > 200 bp) into small noncoding RNAs, which include microRNA (miRNA), small nucleolar RNA(snRNA), small interfering RNA(siRNA), Piwi-interacting RNA(piRNA) and long ncRNA (lncRNA) [41]. Within small ncRNAs, miRNA is a type of endogenous, noncoding small RNA that plays a significant role in modulating gene expression at the posttranscriptional level. *H. pylori* has the ability to disrupt miRNA expression, enabling it to avoid or interfere with host defenses and establish persistence within the gastric environment [42]. Recent research indicates that during antigen recognition, miRNAs can be transferred from T cells to antigen-presenting cells. These transferred miRNAs possess the capability to regulate gene expression in recipient cells, thereby influencing monosynaptic development [43]. In addition to the well-studied miRNAs, exploring the role of lncRNAs and ncRNAs could provide a better understanding of their relevance to *H. pylori*-related diseases. However, the existing mechanisms are currently limited.

### Influence of Exosome-ncRNAs in gastrointestinal diseases

**Fig. 2:**
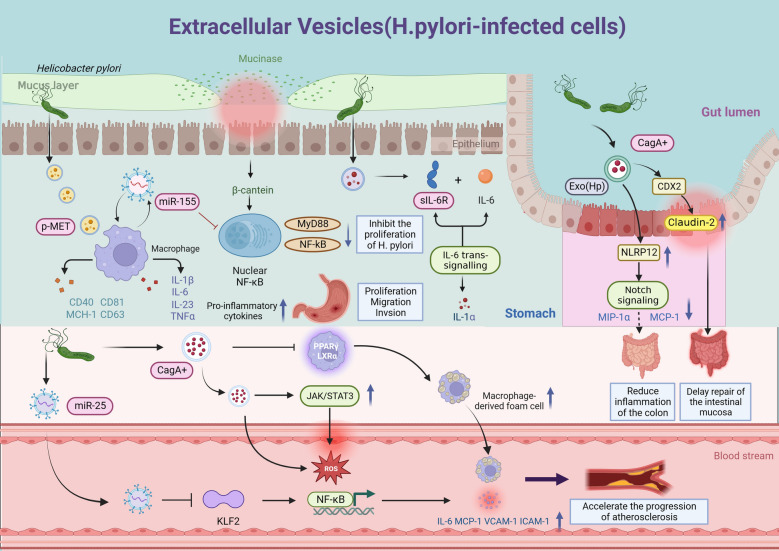
Role of EVs induced by *H. pylori* infection in gastric and extragastric diseases. **A** Following *H. pylori* infection, EVs containing p-MET are released. They are then internalized by macrophages, which release pro-inflammatory cytokines that promote tumour growth and increase cell proliferation, migration and invasion. **B** MiR-155 exosomes from *H. pylori*-infected macrophages increased the production of the inflammatory cytokines IL-23, IL-6, IL-1βand TNF-α, in addition to the cell signalling proteins CD81, CD63, CD40 and MCH-I. Meanwhile, inflammatory signalling pathway proteins such as MyD88 and NF-kappaB have been downregulated in *H. pylori*-infected macrophages due to miR-155 overexpression. **C** *H. pylori*-derived exosomes upregulate the expression of soluble IL-6 receptor in GES-1 human gastric epithelial cells promoting the presentation of the pro-inflammatory cytokine IL-1α via its mediated IL-6 trans signal. **D** Serum exosomes derived from *H. pylori*-positive patients (Exo(Hp)) increased the expression of NLRP12 in intestinal epithelial cells, and NLRP12 reduced the expression of the chemokines MCP-1 and MIP-1α by inhibiting the Notch signalling pathway, ameliorating colitis symptoms. **E** CagA-containing exosomes derived from GES-1 human gastric epithelial cells regulate the expression of the tight junction protein claudin-2 at the transcriptional level via a CDX2-dependent mechanism, delaying the repair of the intestinal mucosa. **F** *H. pylori* induces gastric epithelial cell-derived exosomal miR-25 can enter the circulation and then regulate the NF-kappaB signaling pathway by targeting the transcription factor KLF2, leading to increased expression of ICAM-1, MCP-1, VCAM-1, and IL-6,, and accelerate the progression of atherosclerosis in vascular endothelial cells. **G** CagA in *H. pylori*-infected vascular endothelial cells-derived exosomes mediated reactive oxygen species(ROS) formation deregulates signals activating signal transducer and activator of JAK-STAT3 in endothelial cells, promoting atherogenesis. **H** CagA in exosomes derived from gastric epithelium infected with *H. pylori* induces the formation of macrophage foam cells and promotes atherosclerosis

Recent studies have revealed the implications of several microRNAs (miRNAs) in the host immune responses triggered by *H. pylori*, including miRNA-125, miRNA-146, miRNA-155, miRNA-21, miRNA-221, and the let-7 family. These particular miRNAs serve a regulatory function in the interactions between toll-like receptors (TLRs) and lipopolysaccharides (LPS), as well as their associated downstream pathways, acting as a connecting link connecting gastric inflammation with the development of pre-neoplastic and malignant lesions [44]. For example, miR-155 has emerged as a pivotal component in both innate immunity and the regulation of inflammatory reactions. When stimulated by *H. pylori*, miR-155 becomes activated in gastric mucosa and epithelial cells, resulting in the increased expression of inflammatory cytokines. This enhanced inflammatory response not only inhibits *H. pylori* proliferation but also helps inhibit the development of gastritis [45]. Moreover, Wang J et al. [46] discovered that *H. pylori*-infected macrophages release exosomes rich in levels of miR-155. These exosomes are then taken up and internalized by macrophages. Following overexpression, miR-155 subsequently leads to the downregulation of MyD88 and NF-κB, which are key proteins in the inflammatory signaling pathway in macrophages infected with *H. pylori*. This downregulation effectively inhibits the inflammatory responses mediated by macrophages, thereby promoting their ability to inhibit or eliminate *H. pylori* and prevent *H. pylori*-induced gastritis (Fig. 2). These findings imply that miR-155 could serve as a novel negative regulator and potentially hold therapeutic value in gastrointestinal diseases caused by *H. pylori* infection. However, a more thorough investigation is required to elucidate the precise mechanisms involved.

### Exosome-ncRNAs promotes atherosclerosis

In endothelial cells, multiple miRNAs play a role in regulating essential inflammatory factors. One such miRNA is miR-25, which influences various cellular processes, including proliferation, apoptosis, and cytokinesis [47]. Findings by Qi et al. [48] support the significance of miR-25 in vascular smooth muscle cell (VSMC) proliferation and the development of TNF-induced atherosclerosis. Yao et al. [49] discovered that individuals with coronary heart disease (CHD) and hypertension have an increased risk of heart failure when their levels of miR-19b-5p, miR-221, and miR-25-5p in peripheral blood mononuclear cells are combined. Furthermore, they found a positive correlation between elevated expression of miR-25-5p and the severity of CHD. Li B et al. [50] demonstrated that there were high levels of miR-25 in *H. pylori*-infected patients’ plasma, indicating that *H. pylori* can induce an increase in the levels of exosomal miR-25 through infection of gastric epithelial cells. Next, Li N et al. [51] determined that exosome miR-25 regulates NF-kappaB signaling pathways in atherosclerosis by targeting Kruppel-like factor 2 (KLF2), an inhibitor of vascular inflammation and atherosclerosis. These findings imply that high levels of exosomal miR-25 may increase the potential risk of coronary heart disease in the peripheral blood. Furthermore, it is possible that *H. pylori* exert biological effects on endothelial cells through the miR-25/KLF2 axis (Fig. 2).

Exosome ncRNAs not only influence inflammation but also impact other pathogenic processes related to atherosclerosis. They regulate lipid metabolism, cholesterol homeostasis, endothelial dysfunction, plaque formation, vascular smooth muscle cell behavior, and vascular calcification. Exosome ncRNAs serve as signaling molecules, communicating between various cell types in the arterial wall, thereby exerting either promotional or inhibitory effects on these processes [52–55].

### OMVs-sncRNAs mediated immune escape

Small non-coding RNAs (sncRNAs) produced by bacteria are classified as small regulatory RNAs (sRNAs), a category of post-transcriptional regulators that control gene expression to adapt to varied environmental conditions or influence the virulence genes of pathogenic microorganisms [56]. Bacterial OMVs play a vital role in host–pathogen communication. Recent evidence suggests that OMVs influence host immune responses through the inclusion of different packaged sncRNAs targeting the function of host mRNA [57]. According to Zhang et al. [58], two specific sncRNAs (sR-2509025 and sR-989262) have been proposed to facilitate the delivery of *H. pylori* into host cells, which are abundant in OMVs. These sncRNAs reduce the secretion of IL-8 induced by lipopolysaccharides or OMVs in AGS cells cultured in vitro, thereby facilitating immune evasion. Li et al. [59] further showed that OMV-encapsulated sncRNA is essential in regulating the immune response in aminal hosts infected by *H. pylori*. The results showed that sR-2509025 and sR-989262 stimulated higher serum IgG and IgA production in mice. The levels of vaginal sIgA and gastric sIgA confirmed that the depletion of sncRNAs suppressed the immune response of the host, leading to enhanced mucosal and humoral immunity. These findings provide support for the hypothesis that sncRNAs present in *H. pylori* OMVs play a critical role in directly modulating the host immune response, allowing *H. pylori* to evade the host immune response. However, further research on host–pathogen interactions is warranted.

### Proteins in the exosomes of *H. pylori*-infected Host Cells

Proteins within EVs include those involved in their biogenesis, such as the ESCRT machinery, cytoskeletal components, transmembrane, adhesion, antigen presentation, and other proteins [60]. Increasing evidence suggests that *H. pylori* infection can shift the protein profile of exosomes emitted by a host cell and contribute to various cellular functions. For instance, the exosomal mesenchymal–epithelial transition (MET) factor may be a promising modulator of the protumorigenic role of macrophages in GC pathogenesis. Che Y et al. [61] observed that EVs loaded with phosphorylated mesenchymal–epithelial transition factor (p-MET) are released following *H. pylori* infection. Macrophages ingest them, releasing inflammatory cytokines that stimulate tumor growth, proliferation, invasion, and migration (Fig. 2). To investigate the changes in EVs released from gastric host cells during *H. pylori* infection, González et al. [62] isolated and characterized EVs from *H. pylori*-infected and non-infected human gastric epithelial cells GES-1 (designated as EVHp + and EVHp-, respectively). They demonstrated that extracellular vesicles from *H. pylori*-infected gastric epithelial cells promoted alterations in receptor cells associated with malignancy. These alterations included decreased cell viability, increased IL-23 concentrations, enhanced migration, and transendothelial invasion. In another study, Chen et al. [63] cultured human intestinal epithelial cells with serum exosomes obtained from patients diagnosed with *H. pylori*-positive chronic gastritis. They employed an antibody microarray or PCR array to analyze cytokine and gene expression in signaling pathways. Their findings demonstrated that serum exosomes from *H. pylori*-infected patients with chronic gastritis stimulated the soluble IL-6 receptor in human gastric epithelial cells, resulting in the release of the proinflammatory cytokine IL1-α (Fig. 2). IL1-α is expressed in immune cells, epithelial cells, and stromal cells alike, and it prominently contributes to the development of various human conditions, including inflammation and cancer.

Another research team [64] reported that exosomes derived from *H. pylori* augmented the levels of NLRP12 inflammatory vesicles in intestinal epithelial cells. Furthermore, NLRP12 suppressed the expression of chemokines MCP-1 and MIP-1α by inhibiting the Notch signaling pathway, thereby improving the symptoms of colitis (Fig. 2).

### *H. pylori*-specific proteins in exosomes

Moreover, specific proteins of *H. pylori* can be secreted into host cell-derived exosomes. One such protein is cytotoxin-associated gene A (CagA), which acts as a virulence factor. Upon entry into host cells via the type IV secretory system (T4SS) [65], CagA triggers multiple signal transduction pathways, resulting in alterations in cell structure and an elevated susceptibility to gastrointestinal disorders [66]. Exosomes transport CagA proteins to remote locations from the primary disease, potentially causing extra-gastric diseases. However, the mechanism by which *H. pylori* and its products traverse the epithelial barrier and enter the bloodstream is still under investigation [67].

Shimoda et al. [68] employed liquid chromatography-tandem mass spectrometry (LC–MS/MS) to investigate whether CagA is expressed in exosomes released by CagA + *H. pylori*-infected individuals. They discovered that exosomes originating from CagA-expressing gastric epithelial cells travel through the bloodstream to distant organs and tissues. In a study conducted by Xia X et al. [69], exosomes obtained from CagA + *H. pylori*-infected human gastric epithelial cells, as well as serum exosomes from *H. pylori*-infected individuals and mice, markedly hindered endothelial functions in vitro. This impairment was manifested through reduced migration, tube formation, and proliferation. In *H. pylori*-infected mice, inhibition of exosome release via GW4869 effectively preserved endothelial function. The authors of this study [70] further suggested that CagA + *H. pylori*, as opposed to CagA- *H. pylori*, protects against infection-induced endothelial dysfunction and contributes to the development of atherosclerosis through the generation of ROS via CagA-containing exosomes. Additionally, a recent investigation revealed that CagA delivered by exosomes can deactivate the JAK-STAT3 signaling pathway in endothelial cells, thereby accelerating the inflammatory response or facilitating the production of reactive oxygen species, promoting atherogenesis [71] (Fig. 2). More specifically, Yang S et al. [72] proposed that CagA-positive *H. pylori* infection did not cause atherosclerosis, but it accelerated its progression via exosomes. CagA in *H. pylori*-infected gastric epithelium-derived exosomes inhibits cholesterol transporter protein transcription by downregulating the expression of transcription factors PPARγ and LXRα. This consequently leads to the development of foam cells derived from macrophages and promotes the progression of atherosclerosis (Fig. 2). The findings of this study provide evidence that the primary virulence factor of *H. pylori*, known as CagA, may play a role in the formation of vascular lesions by facilitating the transmission of exosomes. This discovery presents a novel mechanism for elucidating the extra-gastric manifestations of *H. pylori* disease.

Additionally, Guo et al. [67] discovered that CagA-positive *H. pylori* strains disrupted the integrity of the intestinal mucosal barrier and heightened the damage inflicted on the intestinal epithelium by IFN-gamma. These effects were facilitated by exosomes that functioned as mediators and involved CagA. Specifically, CagA upregulated the transcriptional expression of claudin-2 through a CDX2-dependent mechanism, thus delaying the recovery of colitis-damaged mucosa in vitro (Fig. 2).

### Proteins in the *H. pylori*-derived OMVs

**Fig. 3:**
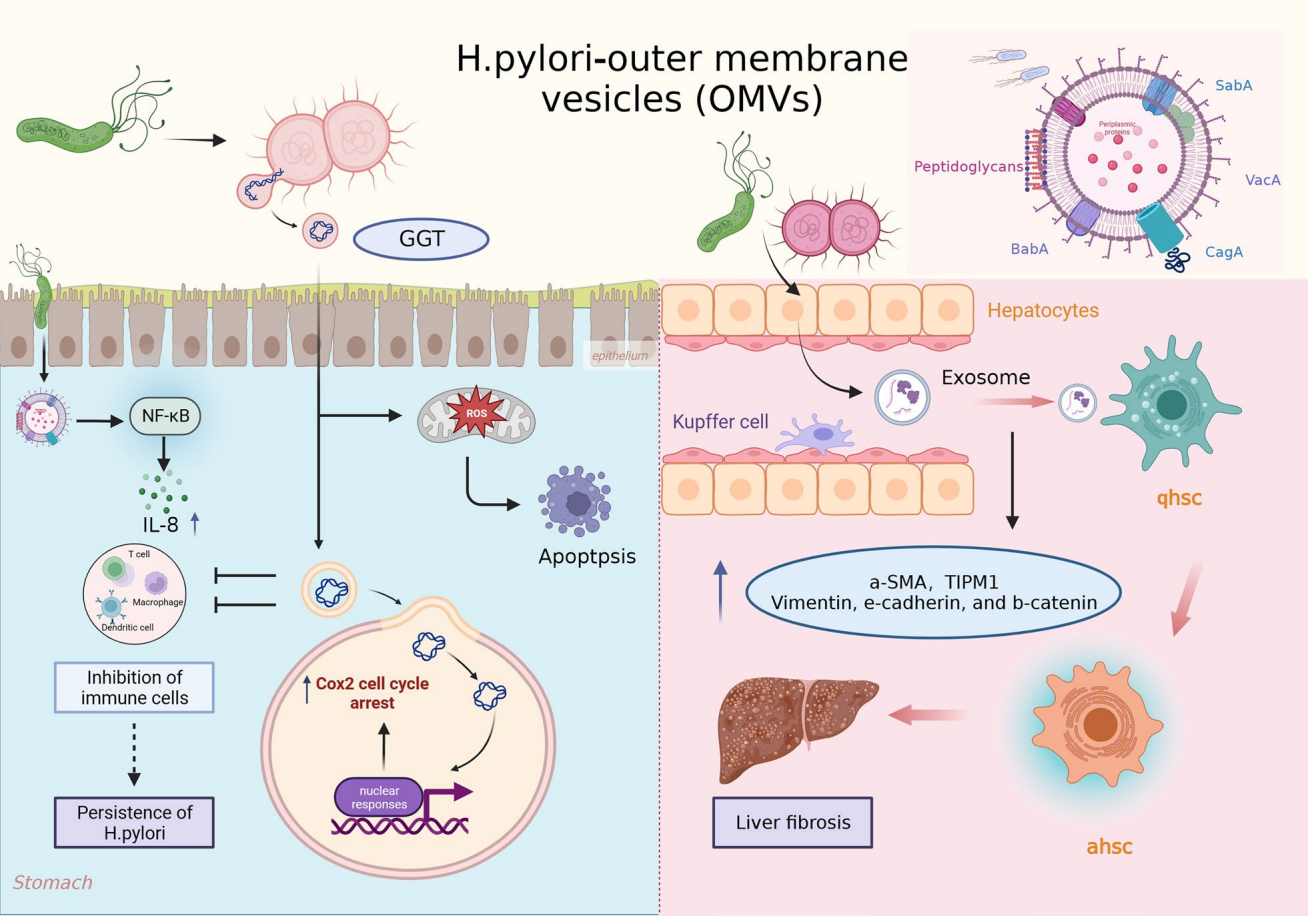
Role of *H. pylori*-OMVs in gastric and extragastric diseases. **A** *H.pylori*-derived OMVs may contribute to the inflammation of gastric epithelial cells through the induction of IL-8 production by NF-kappaB activation. **B** *H. pylori* derived gamma-glutamyl transpeptidase(GGT) induces the production of ROS and then promotes cell apoptosis; In addition, by inhibiting T-cell mediated immunity and dendritic cell differentiation, GGT could induce cell cycle arrest via the nuclear response and immune tolerance. **C** In addition, H.pylori-derived OMV upregulates the expression of hematopoietic stem cell activators and fibrosis markers in exosomes secreted by hepatocytes. Further activation of hepatic stellate cells (ahsc) promotes the progression of liver fibrosis

Several studies have demonstrated that OMVs derived from *H. pylori*, once internalized, can induce the secretion and release of pro-inflammatory cytokines and chemokines. This triggers an inflammatory response and recruits immune cells to the site of infection. Choi HI and coworkers [73] initially found that gastric fluids of patients with gastric cancer contained a considerably higher presence of *H. pylori* cells and *H. pylori*-derived OMVs compared to healthy individuals serving as controls. These OMVs from *H. pylori* contribute to a chronic inflammatory state in the gastrointestinal tract and promote persistent *H. pylori* infection. Gastrointestinal OMVs protect pathogens, facilitate infection, impair cell function, and regulate host immune defense by inducing apoptosis in immunosuppressive cytokines interleukin-10 and Jurkat T cells. These cytokines are generated by human peripheral blood mononuclear cells (PBMCs) [74]. GGT acts as a virulence factor, resulting in cell cycle arrest, apoptosis, and necrosis in stomach epithelial cells. It depletes glutathione (GSH), generates reactive oxygen species, and induces apoptosis in oral cavity epithelial cells [75, 76]. Furthermore, GGT fosters immune tolerance by inhibiting T cell-mediated immunity and dendritic cell differentiation, thereby facilitating the persistence and colonization of *H. pylori* [77, 78] (Fig. 3). Later, Choi MS et al. [79] found that OMVs derived from *H. pylori* potentially contribute to the pathogenesis of diverse gastric diseases by inducing IL-8 expression via NF-kappaB activation. Through the isolation of OMVs derived from *H. pylori* using endoscopic biopsy samples obtained from patients with gastric ulcer, gastritis, or gastric cancer, the researchers observed variations in the size and morphology of the vesicles across different disease groups. In comparison to healthy controls and patients with gastric ulcers, individuals with gastric cancer displayed significantly elevated IL-8 production and NF-kappaB activation (Fig. 3).

It is important to highlight that *H. pylori*-OMVs can induce inflammation in sites beyond the stomach. Their potential to regulate neutrophil migration indicates an additional mechanism that could contribute to a pro-inflammatory milieu. Nonetheless, additional research is required to fully understand the implications of this finding [80]. Recently, Zahmatkesh ME et al. [81] highlighted the relevance of newly discovered virulence factors, including OMVs. They observed that exosomes derived from OMV-contaminated hepatocytes showed increased expression of markers that activate hepatic stellate cells (TIPM1 and a-SMA) and markers associated with fibrosis (e-cadherin, vimentin, and b-catenin) compared to untreated hepatic stellate cells (Fig. 3). These findings propose a potential contribution of *H. pylori* OMVs in modifying hepatocellular exosomes, with potential implications for stellate cell activation and liver fibrosis progression. Additionally, Xie J et al. [82] demonstrated that OMVs appear to communicate with the communication pathway between the brain and gut in *H. pylori* infection by modulating the physiological functions of glial cells and neurons, and may worsen Alzheimer’s disease pathology. This study further supports the concept that OMVs derived from *H. pylori* are associated with the pathogenesis of diseases outside the gastrointestinal tract and suggests that further research is warranted to determine the exact mechanism of *H. pyl*ori’s extra-gastrointestinal manifestations and pathogenesis.

### Application of extracellular vesicles

EVs have emerged as promising candidates for liquid biopsy of tumors due to their unique expression patterns and the consistent composition they exhibit. To detect methylation, Yamamoto et al. [83] employed EVs derived from gastric cancer cell strains, normal gastric cells, and gastric juice. They identified elevated levels of methylation in the BARHL2 gene in gastric juice from early gastric cancer patients and gastric cancer cell strains, with a decrease observed in patients diagnosed with regular gastritis or atrophic gastritis. Assessing the methylation of BARHL2 in EV DNA derived from gastric fluid could serve as a potential biomarker for monitoring the early stages and progression of gastric cancer. Fu and colleagues [84] found that the level of TRIM3 protein in exosomes isolated from the serum of gastric cancer subjects was significantly lower compared to healthy volunteers, suggesting that TRIM3 expression in exosomes isolated from the serum could serve as a potential predictive biomarker for gastric cancer. In another study, Shi et al. [85] utilized exosomal RNA in their pulldown analysis, revealing that miR-1246, previously identified as significantly upregulated in the plasma of gastric cancer patients, is encapsulated in exosomes with the involvement of HuR. This finding offers a justification for considering miR-1246 as a potential biomarker for the specific condition.

Characterizing the role of EVs in the autoimmune microenvironment may aid in identifying new therapeutic targets. First, considering their ability to activate host immune responses such as T-cell responses, B-cell antibody secretion, and inflammatory responses, EVs have been proposed as a potential vaccine. Meanwhile, under the premise of effectively inducing protective immunity, EVs can reduce bacterial load and improve functional immune efficiency [86]. Song Z et al. [87] examined the viability of naturally secreted OMVs in gram-negative bacteria as immunogens with clinical effectiveness. Their findings revealed that OMVs not only boosted humoral and mucosal immunity but also had significant inhibitory effects on *H. pylori* colonization, ultimately leading to the crucial outcome of promoting *H. pylori* eradication. As a result, OMVs could be used as an adjuvant in developing a new generation of vaccines against *H. pylori* infection. Li Y et al. [88] created a powerful biomimetic nanomedicine by coating drug-loaded polymer micelles with bacterial OMVs to achieve effective cancer immunotherapy and inhibit metastatic spread. In vivo, OMV anti–programmed cell death protein 1 (PD-1) promotes tumor immune cell infiltration and the antitumor immune response. Its high accumulation at tumor sites and effective binding with PD-L1 on tumor cells eventually block the PD1/PD-L1 inhibitory axis by exhausting PD-L1 on tumor cells, leading to a higher antitumor response. This study demonstrates the potential of OMVs as immunotherapy drugs that can comprehensively regulate the tumor immune microenvironment and significantly improve therapeutic antitumor efficacy.

## Conclusions and future research

Host cells infected with *H. pylori* release EVs and OMVs, which play a crucial role in facilitating intercellular communication, tumor progression, vascular function, and immunity in the development of *H. pylori*-related diseases. The characteristics of *H. pylori* EVs enable them to breach the stomach wall and access the bowel and bloodstream, making them potential key players in *H. pylori* pathobiology and the development of extra-gastric manifestations as vectors of virulence factors. Although some progress has been made in studying EVs in *H. pylori* infection, several issues remain unresolved. Given that a majority of EVs studies have employed cell culture models, it is crucial to ascertain the applicability of in vitro observations to animal models that mimic cell-specific knockdown of exosome release. Further investigation is needed to understand the dual role of EVs in *H. pylori* infection and the interaction between OMVs secreted by *H. pylori* and host cell exosomes. Additionally, more research is needed to explore the time-dependent variability of EV charge during disease progression and its impact on cell interface markers. The emergence of multidrug-resistant microorganisms and the post-antibiotic era have underscored the need for new strategies in controlling infectious and contagious diseases. Advancements in scientific and engineering technologies related to intracellular vesicles could serve as an invaluable tool in combating contagious pathogens.

## Data Availability

All relevant data were included in the paper.

## Abbreviations

EV: Extracellular vesicles
H. pylori: Helicobacter pylori
MALT: Mucosa-associated lymphoid tissue
AS: Atherosclerosis
COPD: Chronic obstructive pulmonary disease
NAFLD: Nonalcoholic fatty liver disease
IBD: Inflammatory bowel disease
AD: Alzheimer's disease
ncRNA: Non-coding RNA
ISEV: International Society for Extracellular Vesicles
MVs: Microvesicles
ABs: Apoptotic bodies
ESCRT: Endosomal sorting required for transport
ILVs: Intraluminal vesicles
MVBs: Multivesicular bodies
bEVs: Bacterial extracellular vesicles
OMVs: Outer membrane vesicles
VacA: Vacuolating cytotoxin A
snRNA: Small interfering RNA
lncRNA: Long ncRNA
siRNA: Small interfering RNA
miRNA: MicroRNA
piRNA: Piwi-interacting RNA
VSMC: Vascular smooth muscle cell
CHD: Coronary heart disease
KLF2: Kruppel-like factor 2
MET: Mesenchymal–epithelial transition
CagA: Cytotoxin-associated gene A
T4SS: Type IV secretory system
LC–MS/MS: Liquid chromatography–tandem mass spectrometry
PBMCs: Peripheral blood mononuclear cells
GGT: Gamma-glutamyl transpeptidase
GSH: Depletion of glutathione
PD-1: Programmed cell death protein 1
